# Buffering and Adaptive Coding for Flooding with Randomized Network Coding on Multi-Hop Wireless Broadcasting

**DOI:** 10.3390/s26051594

**Published:** 2026-03-03

**Authors:** Youji Fukuta, Yoshiaki Shiraishi, Masanori Hirotomo, Masami Mohri

**Affiliations:** 1 Faculty of Informatics, Cyber Informatics Research Institute, Kindai University, Higashiosaka-shi 577-8502, Japan; 2 Graduate School of Engineering, Kobe University, Kobe-shi 657-8501, Japan; 3 Faculty of Science and Engineering, Saga University, Saga-shi 840-8502, Japan

**Keywords:** adaptive coding, broadcast flooding, buffer management, randomized network coding, wireless ad hoc networks

## Abstract

Broadcast-based flooding in wireless ad hoc networks is subject to the broadcast storm problem, characterized by excessive transmissions, collisions, and link losses. While randomized network coding (RNC) enhances resilience against packet losses, efficient buffer management and adaptive transmission strategies are essential. This paper proposes novel buffering mechanisms and adaptive coding strategies to improve data unit reception rates in RNC-based broadcast flooding. Our buffering mechanism combines Last-In-First-Out (LIFO) and Least Recently Used (LRU) discard policies. When buffers are full, it prioritizes the discarding of stale, incomplete buffers based on elapsed time since the last coded block arrival, thereby overcoming First-In-First-Out (FIFO) limitations that prematurely discard buffers before sufficient coded blocks have accumulated. Our adaptive coding dynamically adjusts transmitted coded packets based on data unit duplication rates without inter-node coordination, reducing blocks during high duplication and increasing them under difficult reception conditions. Simulation experiments using OMNeT++ and INET framework for Vehicular Ad Hoc Networks demonstrate that LIFO+LRU buffering significantly increases the received data units and prevents redundant reception, while adaptive coding further improves reception rates under challenging conditions.

## 1. Introduction

Mobile ad hoc networks (MANETs) are distributed wireless networks in which mobile devices communicate directly with each other without relying on fixed infrastructure such as base stations or access points. Unlike traditional wireless networks, MANETs do not require a fixed communication infrastructure. Each node (device) operates autonomously, serving as both an end system and a router. Networks are formed automatically and dynamically reconfigured in response to node participation and departure. No central administrator or preconfiguration is required, allowing networks to be established instantly as needed. Network topology changes frequently due to node mobility, failures, and network joins and departures. This dynamic nature necessitates adaptive routing protocols. For nodes beyond direct communication range, multi-hop communication is conducted through relay nodes. Each node extends the communication range by forwarding packets for other nodes. Mobile devices have constraints such as limited battery power, computational capability, memory, and bandwidth.

Wireless broadcast is a fundamental communication method utilized for several important purposes in MANETs. A critical application is route discovery in routing protocols. In protocols such as AODV and DSR, sender nodes broadcast Route Request (RREQ) messages, which are rebroadcast by relay nodes to discover routes to destination nodes. When link breaks occur, error notifications are propagated to affected nodes to trigger route reconstruction. In many routing protocols, nodes periodically broadcast hello messages to confirm the presence of neighboring nodes and maintain link status. In proactive protocols such as the Optimized Link State Routing (OLSR) protocol, topology control messages are periodically disseminated through controlled flooding using multipoint relays (MPRs), enabling all nodes to maintain a consistent view of the network structure. Service-providing nodes may broadcast information about available services (such as file sharing and printers) and available resources (such as CPU, memory, and remaining battery), depending on the application design. This enables other nodes to efficiently discover needed services and resources. In disaster scenarios, emergency alerts and hazard information are rapidly transmitted to all nodes via broadcast. In Vehicular Ad Hoc Networks (VANETs), traffic safety messages and accident information are continuously broadcast. Broadcast mechanisms are often utilized to support multicast group formation and control operations. Nodes equipped with the Global Positioning System (GPS) broadcast location information to enable location-based routing and geocasting. From a security perspective, public keys, intrusion detection alerts, and blacklist information of malicious nodes may be disseminated via broadcast mechanisms depending on the security framework employed.

Flooding [1,2] is the most fundamental information dissemination method in MANETs, distributing information throughout the network by forwarding received packets to all neighboring nodes. In flooding, a sender node broadcasts a packet, and each receiver node, upon first reception, rebroadcasts it to all neighboring nodes. This process repeats, propagating the packet throughout the network. Duplicate received packets are discarded, preventing infinite loops. Simple flooding causes serious problems known as the broadcast storm problem [3], which consists of three main challenges. First is redundancy, where the same packet is repeatedly transmitted from numerous nodes, particularly in high-density networks. Second is contention, where many nodes attempting simultaneous transmission intensify media access contention. Third is collision, where packet collisions frequently occur on wireless channels, increasing reception failure rates.

Methods employing randomized network coding have been proposed to address the broadcast storm problem in flooding over wireless ad hoc networks [4]. With fixed generation size and number of coded packets, transmitting nodes send multiple coded packets. When packet loss occurs on a wireless path connected to a receiving node, decoding becomes possible using coded packets obtained from other wireless paths, reducing the decoding failure probability. In multi-hop wireless networks, assuming combined use of broadcast and unicast, adaptive network coding methods using randomized network coding have been proposed to address packet loss on wireless paths [5]. Each node estimates the link loss rate by receiving unicast ACKs from nodes that received coded packets broadcast by that node. Each node dynamically adjusts the number of generated coded packets (greater than the generation size) according to the link loss rate, maintaining reliability while suppressing excessive redundant transmission. Compared to a fixed redundant packet number, the decoding failure probability is reduced.

We aim to mitigate the impact of the broadcast storm problem (addressing packet loss) in flooding with randomized network coding to achieve multi-hop wireless broadcast, assuming many-to-many communication in mobile ad hoc networks. This paper proposes buffering and adaptive coding to improve the number of data units reaching nodes in flooding with randomized network coding in wireless mobile ad hoc networks.

Our approach introduces two key innovations that address fundamental challenges in RNC-based flooding without requiring inter-node coordination. First, we propose a novel buffer management strategy combining Last-In-First-Out (LIFO) and Least Recently Used (LRU) discard policies. Unlike conventional First-In-First-Out (FIFO) approaches that prematurely discard buffers before sufficient coded blocks accumulate, our LIFO+LRU policy intelligently identifies and discards stale buffers that are unlikely to receive additional blocks while preserving buffers with recent activity. This mechanism significantly increases the probability that nodes can collect a sufficient number of linearly independent coded blocks for successful decoding. Second, we propose adaptive coding that dynamically adjusts the number of transmitted coded packets based on data unit duplication rates observed at each node. When duplication rates are high, indicating significant reception conditions, the mechanism reduces redundant transmissions to conserve bandwidth. Conversely, under difficult reception conditions with low duplication rates, it increases coded packet transmission to enhance reliability. Crucially, this adaptation operates autonomously at each node without requiring unicast acknowledgments or coordination messages, making it practical for dynamic mobile environments where topology changes frequently.

Through simulation experiments in Vehicular Ad Hoc Networks using OMNeT++ and the INET framework, we demonstrate the effectiveness of our proposals. Our LIFO+LRU buffering policy substantially increases the number of accepted data units compared to FIFO and standard LIFO policies while preventing redundant reception of duplicate data units. The adaptive coding mechanism further improves reception rates, particularly for data units traversing multiple hops. The experimental results confirm that combining both mechanisms enables RNC-based flooding to achieve significantly higher data unit delivery rates in mobile ad hoc networks experiencing the broadcast storm problem, without imposing additional coordination overhead or requiring modifications to the existing network infrastructure.

Section 2 presents existing research related to flooding with randomized network coding in wireless mobile ad hoc networks to clarify differences from our approach. Section 3 explains the broadcast storm problem that occurs in flooding via wireless broadcast to clarify the problem we focus on. Section 4 explains randomized network coding, flooding via wireless broadcast using it, and its effects to clarify the flooding with randomized network coding we assume. Section 5 explains the operation of flooding that divides and reconstructs data units and the operation of flooding, applying randomized network coding to it, to clarify the flooding protocol via wireless broadcast. Section 6 presents a buffering model for flooding with randomized network coding that we assume, and explains three buffer discard policies, including our proposal. It also explains our proposed adaptive coding that varies the number of coded packets in flooding with randomized network coding that we assume. Section 7 explains the implementation of flooding that divides and reconstructs data units for the OMNeT++ Flood layer and network layer, the implementation of flooding applying randomized network coding to it, and the implementation of flooding applying our proposed buffering and adaptive coding to it. Section 8 presents simulation experiments using OMNeT++ and their results, providing a discussion. First, it explains experiments comparing flooding with division and reconstruction and flooding with randomized network coding. It also explains experiments comparing cases applying our proposed buffering versus other buffering in flooding with randomized network coding. Furthermore, it presents experiments comparing scenarios with and without the proposed adaptive coding in flooding with randomized network coding, while applying the proposed buffering mechanism. Finally, Section 9 summarizes this research and addresses future challenges and limitations of this study.

## 2. Related Research

In broadcast flooding, for many-to-many communication in wireless ad hoc networks, a method has been proposed that estimates neighboring node mobility based on packet reception status and adaptively applies network coding while maintaining a fixed generation size throughout the network [6]. Neighboring node mobility is calculated as a topology value, and transmission mode is switched between coded transmission and uncoded transmission (simple broadcast) based on comparison with a threshold value. A method introducing Random Linear Network Coding (RLNC) to address the dynamic network topology in vehicle-to-vehicle (V2V) communication on two-way lanes exists [7]. A model is constructed where multiple vehicles (same direction and opposite direction) serve as information sources transmitting to a single sink (receiving vehicle). RNC enables encoding and decoding without prior knowledge of network structure, and the lower bound of the generation probability is theoretically derived. Simulations evaluate delay distribution with respect to variations in packet loss rate and batch size, demonstrating significant delay improvement compared to non-coding methods. An experimental protocol “Tetrys: An On-the-Fly Network Coding Protocol” exists for packet loss recovery in communications requiring real-time performance [8]. While conventional block-based FEC methods require round-trip time (RTT) for loss recovery, Tetrys enables immediate recovery independent of RTT by utilizing elastic encoding windows and generating successive coded packets through a linear combination of unacknowledged source packets. The receiver provides unicast feedback on reception status, and the sender updates the window accordingly, providing resilience to feedback delay and loss. In point-to-point wireless links in Ocean Mobile Internet of Things (OM-IoT) environments, adaptive network coding methods combining sliding window control and reinforcement learning exist that dynamically adjust the generation size and number of coded packets in randomized network coding [9]. Based on unicast acknowledgments (ACKs), channel erasure probability and decoding probability are estimated. The sliding encoding window size, sliding rules, and coding coefficients are dynamically adjusted using reinforcement learning algorithms (Q-learning) and extended greedy strategies. Improvements in packet delivery rate, retransmission rate, redundant transmission rate, and convergence speed of decoding probability have been confirmed, surpassing conventional non-coding and fixed coding methods.

Table 1 summarizes the key characteristics of related work and our approach.

We propose buffering and adaptive coding to mitigate the impact of the broadcast storm problem (addressing packet loss) in flooding with randomized network coding to achieve multi-hop wireless broadcast, assuming many-to-many communication in mobile ad hoc networks. The differences from this research are as follows. The method by Yoshida et al. [6] performs adaptive coding that controls switching between sending coded packets and sending original packets using neighboring node mobility information in flooding with randomized network coding to achieve multi-hop wireless broadcast. It is considered that our proposed buffering and adaptive coding can be applied to this approach. The method by Zhang et al. [7] treats the relationship between sources and destinations as many-to-one and does not assume flooding via wireless broadcast. While they theoretically derive the probability that a sink receives a sufficient number of independent packets when encoding packet groups with a fixed batch size, evaluation when actually operating as a protocol is considered necessary. Tetrys [8] requires unicast feedback for adaptive coding and cannot be directly applied to flooding via wireless broadcast. Since it assumes the use of random coding, there may be room for applying our method in relation to the generation size and the number of coded packets. The method by Zhang et al. [9] performs adaptive coding that varies the generation size and number of coded packets in point-to-point wireless links. Unicast ACK responses between nodes are required for adaptive coding. It is considered that there may be room for applying our buffering method. The contributions of this research are as follows. As OMNeT++ protocols, we implemented flooding with division and reconstruction, flooding with randomized network coding, and flooding with randomized network coding incorporating the proposed buffering and adaptive coding, including processing delays and collision avoidance, enabling simulation experiments. In flooding with randomized network coding via wireless broadcast, we present FIFO and LIFO buffer discard policies, propose a novel LIFO+LRU buffer discard policy, and develop adaptive coding that adjusts the number of coded blocks. Through simulation experiments in VANETs (Vehicular Ad Hoc Networks), we confirm the effectiveness of each approach and demonstrate the validity of the proposed buffering and adaptive coding.

## 3. Broadcast Storm Problem in Wireless Broadcast Flooding

Flooding is the process of broadcasting a packet to all nodes within the radio wave range, after which receiving nodes rebroadcast the packet, thereby distributing the same information to unspecified nodes within the network. Flooding can also be used to send packets to a specific node not listed in the node’s own routing table, rather than sending to unspecified nodes. In this case, once the message reaches the destination node, that node does not forward it. The number of forwarding attempts may also be limited by Time To Live (TTL).

In flooding, a node that receives a new packet must broadcast it only once and forward it to neighboring nodes. This is necessary because the node cannot recognize whether all neighboring nodes have already received the packet. Flooded packets contain data such as sender node ID and sequence number that distinguish them from other flooded packets, and receiving nodes store this information locally. Using this information, if a node receives a packet with the same content for the second or subsequent time, it does not forward it.

An advantage of flooding is that it requires no control information and is easy to control. A disadvantage is that unnecessary broadcasts can strain network bandwidth. Furthermore, while normal unicast communication involves retransmission if an Acknowledgement (ACK) packet is not received, broadcast communication does not involve ACK packet replies or retransmissions, which can result in unreliable communication. Since many unicast protocol methods in ad hoc networks use flooding to exchange routing information, it is necessary to improve the efficiency and reliability of flooding.

A pioneering study exists that systematically analyzes the broadcast storm problem, an essential challenge arising in wireless broadcast communication in mobile ad hoc networks (MANETs) [3]. The study theoretically and through simulation clarifies that simple flooding methods cause redundant retransmissions, severe collisions, and increased communication delay as node density increases. Flooding is used in Route Request (RREQ) messages for searching destination nodes in Ad Hoc On-Demand Distance Vector (AODV) [10], a typical routing protocol for ad hoc networks, and in Topology Control Messages (TC messages) for sharing network topology information in OLSR [11].

While flooding can achieve a high packet delivery rate, it has the disadvantage of causing a large number of redundant packet transmissions because all nodes broadcast. This is called the broadcast storm problem because one broadcast transmission triggers subsequent broadcast transmissions. Various improved flooding algorithms have been proposed to mitigate the broadcast storm problem [3,12,13,14]. One of these is probability-based flooding, which probabilistically broadcasts packets at relay nodes [12,13]. OLSR [11], a routing protocol mentioned above, selects nodes called a multipoint relay set (MPR set) to perform flooding efficiently and restricts the number of nodes that rebroadcast packets.

## 4. Flooding with Randomized Network Coding

In flooding via wireless broadcast, we focus on flooding with randomized network coding to address packet loss caused by broadcast storms. Randomized network coding enables decoding using coded packets obtained from multiple wireless paths when packet loss occurs, thereby reducing decoding failure probability and improving resilience against the broadcast storm problem.

### 4.1. Randomized Network Coding

Two types of network coding exist: physical layer network coding, in which signals from multiple transmitting nodes are combined at the analog level [15], and network layer network coding, in which multiple packets represented as bit sequences are encoded into a single packet [16].

Linear network coding [17] is an encoding scheme for network coding. Each packet is treated as an element of a Galois field, and encoding is carried out using linear operations. Multiple received packets are linearly combined into a single coded packet using coding vectors. The number of packets combined per encoding operation is called the generation size (*N*). Coding vectors must be pre-assigned to each node before encoding takes place.

Pre-assigning coding vectors to every node is impractical in ad hoc networks, where nodes frequently join or leave, and the topology changes dynamically, making centralized assignment infeasible. Randomized network coding [18,19] overcomes this by allowing each node to select its coding vector independently at random.

Early network coding research addressed theoretical aspects such as capacity achievability but left implementation issues—including computational cost, control overhead, and packet-loss resilience—largely unexplored. Random Linear Network Coding (RLNC) [20] addresses these issues by having each node independently encode and forward packets as random linear combinations over a finite field. A receiver can decode the original data once it obtains a sufficient number of linearly independent coded packets, without explicit path control or retransmission control.

**Figure 1:**
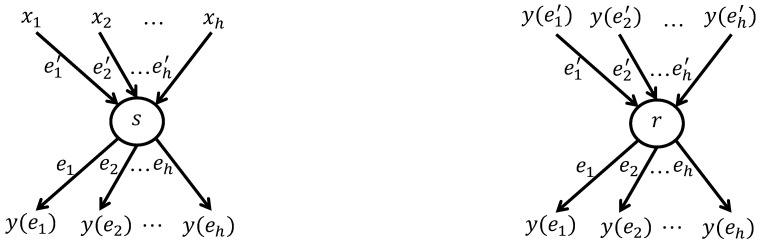
Encoding at the sender and decoding/re-encoding at the receiver.

Figure 1 shows encoding at the sender and decoding/re-encoding at the receiver.

Let (V,E) be an acyclic graph with unit-capacity edges, s∈V the source, and T⊆E the set of receivers. The broadcast capacity *h* equals the minimum edge-cut between *s* and any receiver. Each edge e∈E from node v=in(e) carries y(e), a linear combination of symbols on edges entering *v*, namely $y\left(e\right)=\sum_{e^{\prime }:\mathrm{out}\left(e^{\prime }\right)=v}m_{e}\left(e^{\prime }\right)y\left(e^{\prime }\right)$.

The local encoding vector $m\left(e\right)={\left[m_{e}\left(e^{\prime }\right)\right]}_{e^{\prime }:\mathrm{out}\left(e^{\prime }\right)=v}$ describes the encoding at node *v* on edge *e*. For the source *s*, artificial edges $e_{1}^{\prime },\ldots ,e_{h}^{\prime }$ are introduced carrying source symbols $y\left(e_{i}^{\prime }\right)=x_{i},i=1,\ldots ,h$.

By induction, $y\left(e\right)=\sum_{i=1}^{h}g_{i}\left(e\right)x_{i}$ on any edge *e*, where the *h*-dimensional coefficient vector $g\left(e\right)=[g_{1}\left(e\right),\ldots ,g_{h}\left(e\right)]$ is computed recursively as $g\left(e\right)=\sum_{e^{\prime }:\mathrm{out}\left(e^{\prime }\right)=v}m_{e}\left(e^{\prime }\right)g\left(e^{\prime }\right)$, with $g(e_{i}^{\prime })$ initialized to the *i*-th unit vector. The vector g(e) is called the global encoding vector of edge *e*.

(1) $$\left[\begin{array}{c} y(e_{1})\\ \vdots \\ y(e_{h}) \end{array}\right]=\left[\begin{array}{ccc} g_{1}\left(e_{1}\right) & \ldots & g_{h}\left(e_{1}\right)\\ \vdots & \ddots & \vdots \\ g_{1}\left(e_{h}\right) & \ldots & g_{h}\left(e_{h}\right) \end{array}\right]\left[\begin{array}{c} x_{1}\\ \vdots \\ x_{h} \end{array}\right]=G_{t}\left[\begin{array}{c} x_{1}\\ \vdots \\ x_{h} \end{array}\right]$$

A receiver *t* obtaining symbols on *h* or more edges $e_{1},\ldots ,e_{h}$ can recover $x_{1},\ldots ,x_{h}$ provided the matrix $G_{t}$ formed by global encoding vectors $g\left(e_{1}\right),\ldots ,g\left(e_{h}\right)$ has rank *h*. This holds with high probability when coding vectors are chosen randomly over a finite field of sufficient size.

Each scalar symbol y(e) is extended to a vector $y\left(e\right)=[y_{1}\left(e\right),y_{2}\left(e\right),$$\ldots ,y_{N}\left(e\right)]$, and the same linear combination rule applies: $y\left(e\right)=\sum_{e^{\prime }:\mathrm{out}\left(e^{\prime }\right)=v}m_{e}\left(e^{\prime }\right)y\left(e^{\prime }\right)$ over the vectors $y(e^{\prime })$ entering v=in(e).

Each source symbol $x_{i}$ on artificial edge $e_{i}^{\prime }$ becomes a vector $x_{i}=\left[x_{i,1},x_{i,2},\ldots ,x_{i,N}\right]$, so any receiver can reconstruct the *h* source vectors $x_{1},\ldots ,x_{h}$ from any *h* received packets.

(2) $$\left[\begin{array}{c} y(e_{1})\\ \vdots \\ y(e_{h}) \end{array}\right]=\left[\begin{array}{cccc} y_{1}\left(e_{1}\right) & y_{2}\left(e_{1}\right) & \ldots & y_{N}\left(e_{1}\right)\\ \vdots & \vdots & \ddots & \vdots \\ y_{1}\left(e_{h}\right) & y_{2}\left(e_{h}\right) & \ldots & y_{N}\left(e_{h}\right) \end{array}\right]=G_{t}\left[\begin{array}{c} x_{1}\\ \vdots \\ x_{h} \end{array}\right]=G_{t}\left[\begin{array}{cccc} x_{1,1} & x_{1,2} & \ldots & x_{1,N}\\ \vdots & \vdots & \ddots & \vdots \\ x_{h,1} & x_{h,2} & \ldots & x_{h,N} \end{array}\right]$$

Every packet on edge *e* carries the *h*-dimensional global encoding vector g(e). Receivers can therefore obtain all encoding vectors they need directly from the arriving packets.

In practice, the *i*-th unit vector is prepended to the source vector $x_{i},i=1,\ldots ,h$, and each node processes the augmented vectors in the usual way. Any receiver can then retrieve $x_{1},\ldots ,x_{h}$ by applying Gaussian elimination to its *h* received packets.  

(3) $$\begin{array}{c} \left[\begin{array}{ccccccc} g_{1}\left(e_{1}\right) & \ldots & g_{h}\left(e_{1}\right) & y_{1}\left(e_{1}\right) & y_{2}\left(e_{1}\right) & \ldots & y_{N}\left(e_{1}\right)\\ \vdots & \ddots & \vdots & \vdots & \vdots & \ddots & \vdots \\ g_{1}\left(e_{h}\right) & \ldots & g_{h}\left(e_{h}\right) & y_{1}\left(e_{h}\right) & y_{2}\left(e_{h}\right) & \ldots & y_{N}\left(e_{h}\right) \end{array}\right]\\ =G_{t}\left[\begin{array}{ccccccc} 1 & \; & 0 & x_{1,1} & x_{1,2} & \ldots & x_{1,N}\\ \; & \ddots & \; & \vdots & \vdots & \ddots & \vdots \\ 0 & \; & 1 & x_{h,1} & x_{h,2} & \ldots & x_{h,N} \end{array}\right] \end{array}$$

### 4.2. Flooding with Randomized Network Coding

In conventional simple flooding, the broadcast storm problem occurs as node density increases, including increased retransmissions and frequent collisions. A new broadcast method using randomized network coding has been proposed to improve the efficiency of broadcast communication in dense wireless ad hoc networks [4]. By introducing a method in which each node randomly linearly combines multiple received packets and retransmits them, redundant retransmissions can be suppressed while maintaining a high delivery rate.

**Figure 2:**
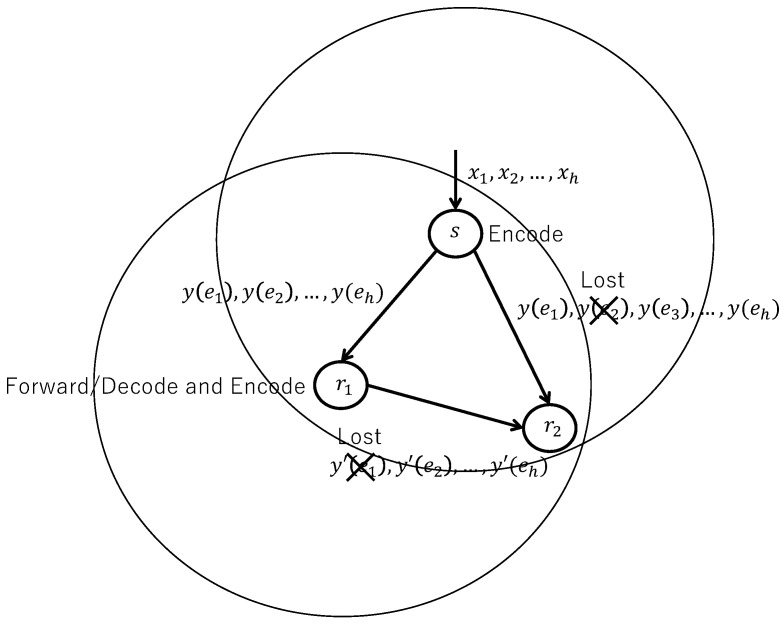
Encoding at the sender and decoding/decoding and encoding at the receiver in flooding with randomized network coding.

Figure 2 shows encoding at the sender and decoding/decoding and encoding at the receiver in flooding with randomized network coding.

In linear network coding, each receiver *r* has to receive *h* linearly independent packets in order to retrieve the original packets. Therefore, the sender *s* divides information into groups with *h* packets and assigns the same sequence number (generation number) to every *h* packets. Then, it encodes the *k*-th (k=1,…,h) packet in each group with the associated coding vector $e_{k}$, where $e_{k}$ denotes the *k*-th unit vector in $GF(2^{m})$, i.e., the *k*-th element in $e_{k}$ is given by the unit element in multiplication and all other elements are given by the zero element. By doing so, neighboring nodes of the sender *s* can decode received packets correctly, a sequence of packets generated by a sender *s*. The sequence number (generation number) increases by one every *h* packets.

Let $h_{r}$ denote the number of neighboring nodes of a receiver *r*. We assume that each receiver *r* knows $h_{t},t\in V_{r}$, where $V_{r}$ denotes a set of neighboring nodes of *r*. Note that this information can be obtained by periodically exchanging hello messages among receiver nodes. If a received packet at a receiver *r* has the same sequence number (generation number) as any of the packets that have already been retrieved, the node *r* discards it. Otherwise, the receiver *r* stores it in the buffer. When the receiver *r* gathers *h* coded packets with the same sequence number (generation number), it tries to decode those in order to retrieve the original packets.

Consider that a receiver *r* tries to decode *h* received packets. If and only if *h* original packets are retrieved successfully, the receiver *r* relays the packets to all its neighboring nodes ${\hat{r}}_{l}\in V_{r},l=1,\ldots ,\left|V_{r}\right|$ as follows. If $h_{{\hat{r}}_{l}}\geq h$ for all *l*, the receiver *r* encodes the retrieved original packets with a new coding vector constructed at the node *r* and transmits the resulting output packet to all the neighboring nodes. On the other hand, if there exists a neighboring node ${\hat{r}}_{l}$ with $h_{{\hat{r}}_{l}}<h$, the receiver *r* transmits *h* packets with unit coding vectors $e_{k},k=1,\ldots ,h$, as the sender *s* does.

The discussion of flooding with random network coding in the following sections is grounded in the method described herein. If a receiving node fails to decode the received packets, it discards all *h* packets. In contrast, when decoding is successful, the node re-encodes the packets and forwards them, irrespective of the number of neighboring nodes. Moreover, if the *h* packets do not arrive within a predefined time interval, the node forwards the received packets to its neighboring nodes without performing decoding.

## 5. Behavior of Wireless Broadcast Flooding

In flooding via wireless broadcast, packets are transmitted consecutively along the same path, with other packets associated with the same set of source vectors $x_{1},x_{2},\ldots ,x_{h}$. All packets associated with the same set of source vectors $x_{1},x_{2},\ldots ,x_{h}$ are said to be in the same generation. The parameter *h* is called the generation size. All packets in the same generation are assigned the same sequence number (generation number).

### 5.1. Behavior of Flooding with Data Unit Fragmentation and Reconstruction

The sender divides the data unit into *h* blocks and assigns the source address and the same sequence number to every packet, including the block and the block ID, and transmits (broadcasts) them in order. To prevent accepting a data unit that has already been transmitted, a new buffer is created, and the original blocks are stored in the buffer.

**Figure 3:**
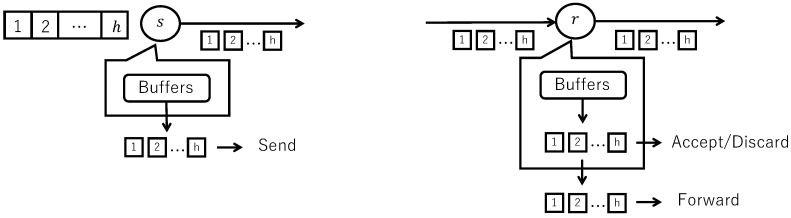
Behavior of flooding with data unit fragmentation and reconstruction.

Figure 3 shows the behavior of flooding with data unit fragmentation and reconstruction.

When a packet including a block arrives, the receiver checks the sender’s address, sequence number, and block ID, and if it is the first time it has arrived, adds the block to the corresponding buffer. When the block belonging to a data unit arrives for the first time, a new buffer is created, and the block is stored in the buffer. When the block that the sender itself sent or the block that it received previously arrives, the receiver immediately discards it.

When the early release timeout has passed, if the TTL is 2 or greater, the receiver decrements the TTL by 1 and forwards (rebroadcasts) the packets, including the blocks, even if they are not all in the buffer. When the receive timeout (longer than the early release timeout) has passed, if all the blocks are in the buffer, they are reconstructed and accepted. If not all the blocks are in the buffer, they are discarded.

### 5.2. Behavior of Flooding with Randomized Network Coding

The sender divides the data unit into *h* blocks and encodes the *k*-th (k=1,…,h) block in each group with the associated coding vector. The sender assigns the source address and the same sequence number to every coded packet, including the coded block and the coding vector, and transmits (broadcasts) them in order. To prevent accepting a data unit that has already been transmitted, a new buffer is created, and the original blocks are stored in the buffer.

**Figure 4:**
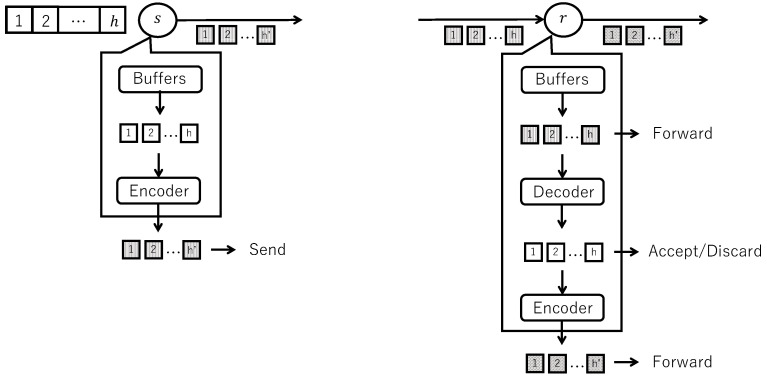
Behavior of flooding with random network coding.

Figure 4 shows the behavior of flooding with random network coding.

When a coded packet including a coded block arrives, the receiver checks the sender’s address, sequence number, and coding vector, and if it is the first time it has arrived, adds the coded block to the corresponding buffer. When the coded block belonging to a data unit arrives for the first time, a new buffer is created, and the coded block is stored in the buffer. When the coded block that the sender itself sent or the coded block that it received previously arrives, the receiver immediately discards it.

If there are *h* linearly independent coded blocks in the buffer, the receiver decodes them into the original blocks. The receiver also uses the original blocks to encode the *k*-th (k=1,…,h) block in each group with the associated coding vector, just like the sender, and re-stores them to the corresponding buffer.

When the early release timeout has passed, if the TTL is 2 or greater, the receiver decrements the TTL by 1 and forwards (rebroadcasts) the coded packets, including the coded blocks. When transferring, if the number of blocks in the buffer is less than the number of blocks (number of encodings), the blocks in the buffer are sent as is. If the number of blocks in the buffer matches the number of blocks (number of encodings), the blocks in the buffer are encoded, and the number of encoded blocks equals the number of encoding blocks, then they are sent. When the receive timeout (longer than the early release timeout) has passed, if all the original blocks are in the buffer, they are reconstructed and accepted. If not all the blocks are in the buffer, they are discarded.

## 6. Buffering and Adaptive Coding Proposals

In flooding with randomized network coding, efficient buffer management and transmission control are essential to improve data unit reception rates under the broadcast storm problem. We propose a buffer discard policy combining Last-In-First-Out (LIFO) and Least Recently Used (LRU) strategies for managing coded blocks received from multiple neighboring nodes. We also propose adaptive coding that dynamically adjusts the number of coded packets based on data unit duplication rates without requiring inter-node coordination.

### 6.1. Buffering on Flooding with RNC

Synchronization of packets associated with the same set of source vectors is an important practical issue for both encoding and decoding nodes. In addition to the source address and sequence number (generation number) in the packet header, each node requires a mechanism to synchronize packet arrivals and departures, which is achieved through buffering.

**Figure 5:**
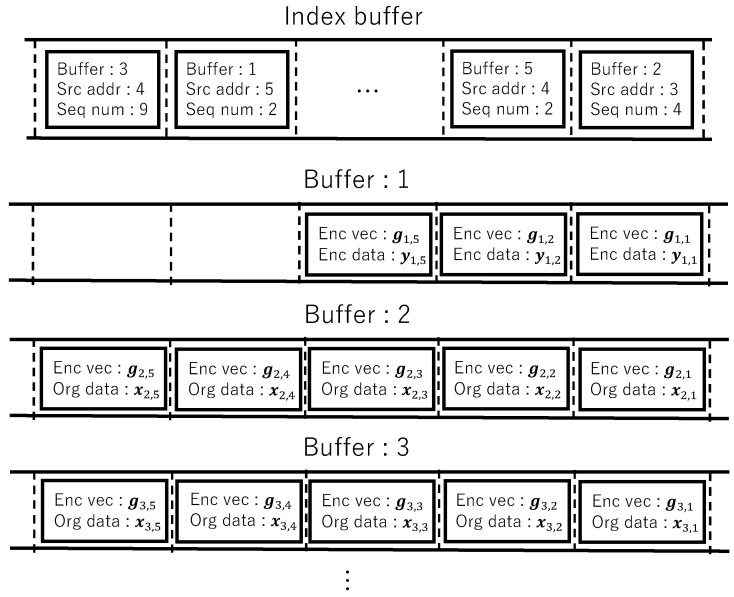
Buffering model on flooding with random network coding.

Figure 5 shows the buffering model on flooding with random network coding.

In the buffering model, when a node transmits a packet or when a packet arrives, the packet is identified by the pair of source address and sequence number (generation number), and the packet data is stored in a single buffer managed by an index buffer.

When a node transmits coded packets, information, including source address, sequence number, and buffer ID, is stored in the index buffer, and a corresponding buffer is created. Original blocks obtained by dividing the data unit are stored in that buffer to prevent re-accepting the packet after transmission. Buffer 3 is an example of this.

When a coded packet of a data unit that does not exist in the index buffer arrives at a node, information including source address, sequence number, and buffer ID is stored in the index buffer, and a corresponding buffer is created. If the coded block and coding vector arrive for the first time, these data are stored in the corresponding buffer. Buffer 1 is an example of this.

When coded blocks are stored in a buffer, and a fixed number (generation size) or more different coded blocks are collected, decoding is applied, a fixed number (generation size) of original blocks are recovered, and these are re-stored in the same buffer. At this time, the data unit is accepted. Buffer 2 is an example of this.

For buffers that have passed the early release timeout, if all original blocks exist in the buffer and these are untransmitted, coded blocks are computed by randomly linearly combining the original blocks in the buffer, and packets containing the coded blocks and coding vectors are generated and forwarded in the number of coded packets. Also, if fewer coded blocks than the generation size exist in the buffer and these are untransmitted, decoding is not applied, and packets containing the coded blocks and coding vectors in the buffer are generated and forwarded.

An index buffer is used to manage buffers that store coded blocks (blocks) of data units. The number of buffers, i.e., the size of the index buffer, is finite, and when the number of buffers reaches the maximum value, buffer discard is performed.

The longer a buffer is retained, the more arriving coded blocks can be received. However, if buffers with insufficient numbers of blocks that are unlikely to receive coded blocks remain, the opportunity to receive new data units decreases. When a node transmits a data unit or when a node receives a coded block of a data unit, the following buffer discard methods are considered.

1. Buffer discard by receive timeout
  When the current number of buffers reaches the maximum number of buffers, buffers satisfying (time of most recent block reception) + (receive timeout) < (current time) are searched. If one applicable buffer is found, the following processing is performed:
  (a) For the buffer in question, the average data unit duplication rate dr is updated using the number of unique coded blocks *x* and the number of duplicate coded blocks *y* (auxiliary information of the buffer). If *x* is greater than or equal to the generation size, dr=(dr+(x+y)/h)/2 is calculated. If *x* is less than the generation size, dr=dr/2 is calculated.
  (b) The buffer in question is discarded.
2. Forced buffer discard
  When the current number of buffers remains at the maximum number of buffers without change, one buffer is discarded according to one of the following policies:
  - FIFO discard policy: The buffer created last is discarded.
  - LIFO discard policy: The buffer created most recently is discarded.
  - LIFO+LRU discard policy: The buffer that has not received coded blocks for the longest time exceeding a threshold time is discarded. If no such buffer exists, the LIFO discard policy is followed.

**Figure 6:**
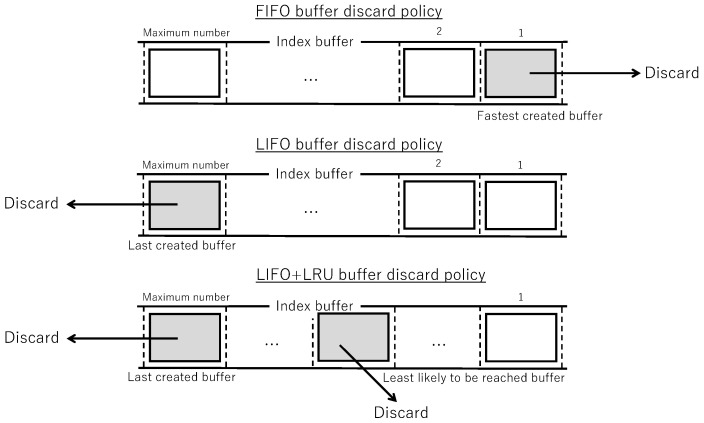
Forced buffer discard on the buffering model.

Figure 6 shows forced buffer discard on the buffering model.

The LIFO+LRU buffer discard policy is presented in algorithmic form in Algorithm 1.

**Algorithm 1** LIFO+LRU buffer discard policy (pseudocode)**Input:**
    bufferList: list of all buffers
    maxBuffers: maximum number of buffers allowed
    thLRU=1.5 s: time threshold
    currentTime: current simulation time
    generationSize (*h*): number of blocks required for decoding 
**Output:**
    Updated bufferList with one buffer discarded 
**Procedure** ForcedBufferDiscard: 
1:    candidateBuffers←∅ 
      // Step 1: Among incomplete buffers, find those exceeding threshold
2:    **for each** buffer in bufferList **do**
3:        numBlocks← number of coded blocks in buffer
4:        **if** numBlocks<generationSize **then**    // Only consider incomplete buffers
5:            lastArrivalTime← time of most recent coded block arrival
6:            elapsedTime←currentTime−lastArrivalTime
7:            **if** elapsedTime>thLRU **then**
8:                Add (buffer,elapsedTime) to candidateBuffers
9:            **end if**
10:       **end if**
11:   **end for** 
      // Step 2: If such buffers exist, discard the one with longest elapsed time
12:   **if** candidateBuffers≠∅ **then**
13:       bufferToDiscard← buffer in candidateBuffers with maximum elapsedTime
14:       Remove bufferToDiscard from bufferList
15:       **return**
16:   **end if** 
      // Step 3: Otherwise, apply LIFO (discard most recently created buffer)
17:   bufferToDiscard← most recently created buffer in bufferList
18:   Remove bufferToDiscard from bufferList


In the FIFO discard policy, when the number of buffers reaches the maximum value, the buffer created last is preferentially discarded. When receiving coded blocks associated with a large number of different data units from multiple neighboring nodes, individual buffers are not retained for a long time, reducing the probability of receiving all coded blocks of a data unit, and the same data unit is accepted and forwarded repeatedly.

In the LIFO discard policy, when the number of buffers reaches the maximum value, the buffer created most recently is preferentially discarded. Regardless of the data unit transmission status from neighboring nodes, individual buffers are retained until the receive timeout is exceeded, increasing the probability of receiving all coded blocks of a data unit and increasing the number of data units reaching individual nodes. Also, by adjusting the receive timeout, it is possible to prevent receiving the same data unit.

Even with the adoption of the LIFO discard policy, among buffers with incomplete data units, there exist those unlikely to receive coded blocks in the future. In the LRU discard policy, among buffers with incomplete data units where the elapsed time since the last coded block arrival exceeds a threshold, the one with the longest elapsed time is judged unlikely to receive coded blocks in the future and is discarded. By combining LIFO and LRU, the probability of receiving all coded blocks of a data unit is further increased, and the number of data units reaching individual nodes increases.

### 6.2. Adaptive Coding on Flooding with RNC

In flooding with randomized network coding, when different coded blocks and coding vectors of a data unit are collected in the generation size at a node, they can be decoded into the original blocks of the data unit, and these can be reconstructed to recover the data unit.

However, when packet loss occurs on a wireless path to a certain node, packet loss is likely to occur similarly on wireless paths between that node and other nodes, and even if coded blocks of the data unit are received via other nodes, it is highly probable that coded blocks will not accumulate to the generation size and cannot be decoded into the original blocks.

When a node receives coded blocks of a data unit, the following adaptive coding that varies the number of coded packets is considered.

1. Encoding and forwarding (early release)
  When a buffer passes the early release timeout, and the minimum value of TTL is greater than 1, the following processing is performed:
  (a) If the average data unit duplication rate dr (calculated during the buffering process) is below or equal to the threshold, the number of coded packets is incremented by +1. If it is above the threshold, the number of coded packets is decremented by −1. The number of coded packets is set with upper and lower limits and varied within that range.
  (b) If all original blocks exist in the buffer and these are untransmitted, coded blocks are computed by randomly linearly combining the original blocks in the buffer, and packets containing the coded blocks and coding vectors are generated and forwarded in the number of coded packets. If fewer coded blocks than the generation size exist in the buffer and these are untransmitted, decoding is not applied, and packets containing the coded blocks and coding vectors in the buffer are generated and forwarded.
2. Decoding
  If a buffer corresponding to the source address and sequence number of the arrived coded block exists, the following processing is performed:
  (a) If the coded block is the same as any coded block in the buffer, the number of duplicate blocks (auxiliary information of the buffer) is incremented by +1. This coded block is discarded, and subsequent processing is skipped.
  (b) If the coded block differs from the coded blocks in the buffer, the coded block is stored in the corresponding buffer. If the number of coded blocks in the buffer matches the generation size, the blocks are decoded into original blocks using Gaussian elimination or similar methods and re-stored in the same buffer. At this time, the data unit is accepted.

**Figure 7:**
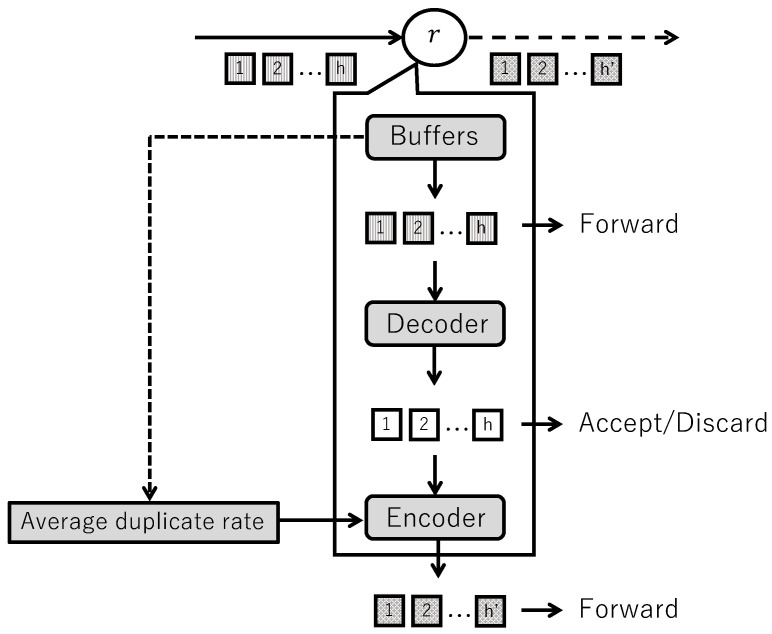
Adaptive coding by varying the number of coded packets.

Figure 7 shows adaptive coding by varying the number of coded packets.

In this adaptive coding, while maintaining a fixed generation size, the average duplication rate of data units arriving at a node is calculated, and the number of coded packets is increased or decreased depending on whether the average duplication rate of data units exceeds a certain value. The average data unit duplication rate is defined as the average number of times complete data units are received in duplicate, and the average duplication rate is updated when buffers that have exceeded the receive timeout are discarded. Updating dr at forced buffer discard events is deliberately avoided, because forced discards may occur after only a short observation window under high packet arrival rates, leading to unstable estimates. By restricting updates to receive timeout-based discards, a stable observation period is guaranteed, yielding reliable duplication rate estimates for adaptive coding.

When a node encodes blocks of a data unit and transmits coded packets containing them, or when a node decodes coded blocks of a data unit, encodes them again, and forwards coded packets containing them, coded blocks are computed by randomly linearly combining the original blocks of the data unit, and packets containing the coded blocks and coding vectors are created in the number of coded packets. The number of coded packets need not match the generation size. By making the number of coded packets larger than the generation size, redundant coded blocks are created, and even if packet loss occurs on a wireless path to a certain node, the probability increases that coded blocks can be obtained up to the generation size from the same wireless path.

In MANETs, since the number of neighboring nodes and the state of wireless paths to those neighboring nodes change frequently when focusing on a certain node, adaptive coding that requires coordination between nodes via wireless unicast becomes complex in processing. This adaptive coding allows each node to independently vary the number of coded packets without coordination between nodes, adjusting the number of coded packets using only wireless broadcast, mitigating the impact even if packet loss occurs on wireless paths, and increasing the number of accepted data units.

## 7. Implementing Protocol in OMNeT++

To validate the effectiveness of the proposed buffering and adaptive coding, flooding protocols via wireless broadcast were implemented for the discrete event simulator OMNeT++ and the INET framework. OMNeT++ provides comprehensive wireless network simulation capabilities with modular architecture, enabling protocol implementation at the network layer for realistic evaluation in mobile ad hoc networks.

### 7.1. OMNeT++ and INET Framework Versions

The implementation uses OMNeT++ 6.2.0 [21] and the INET framework 4.5.4 [22] for simulating many-to-many communication scenarios in wireless broadcast environments.

### 7.2. Implementation of Flooding Protocol Variants

TrafficGen.h, TrafficGen.cc, and TrafficGen.ned in the application layer are used. Three flooding protocol variants were implemented by modifying Flood.h, Flood.cc, and Flood.ned in the Flood layer, and Chunk and Tag in the network layer.

For Plain Flooding with data unit fragmentation and reconstruction, the following information was additionally defined: symbol length symbolLen, number of blocks (number of divisions) numBlock, header length headerLen, payload length payloadLen, processing delay procDelay, early release timeout relTimeout, receive timeout recvTimeout, and average transmission interval of data unit block set blockTime×1.7.

For Plain Flooding with RNC that fragments and reconstructs data units, the following information was additionally defined: symbol length symbolLen, generation size (number of blocks) numBlock, header length headerLen, payload length payloadLen, processing delay procDelay, early release timeout relTimeout, receive timeout recvTimeout, and average transmission interval of data unit block set blockTime×1.7.

For Plain Flooding with RNC, including our proposed buffering and adaptive coding that fragments and reconstructs data units, the following information was additionally defined: symbol length symbolLen, generation size (number of blocks) numBlock, header length headerLen, payload length payloadLen, processing delay procDelay, early release timeout relTimeout, receive timeout recvTimeout, average transmission interval of data unit block set blockTime×1.7, buffer discard policy discardPolicy, time threshold for applying LRU thLRU, upper limit uB and lower limit lB of the number of coded packets for adaptive coding, and duplication rate threshold dTh for adaptive coding.

### 7.3. Packet Format

**Figure 8:**
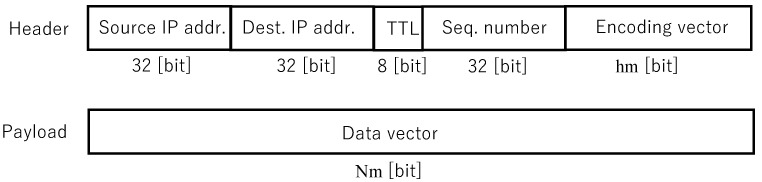
Packet format for flooding with RNC.

Figure 8 shows the packet format for flooding with RNC.

The network layer header stores source IP address (four bytes), destination IP address (four bytes), TTL (one byte), sequence number (four bytes), and encoding vector (*h* bytes in length). The payload stores a data vector (*N* bytes) as a coded block. The symbol length symbolLen is eight bits. *h* and *N* are set within a range that does not exceed the network layer MTU.

In Plain Flooding with fragmentation and reconstruction, the encoding vector length h=1 byte is used, and this area is used as a block identifier. In Flooding with RNC with fragmentation and reconstruction, the encoding vector also serves as a block identifier.

### 7.4. Packet Transmission Timing

**Figure 9:**
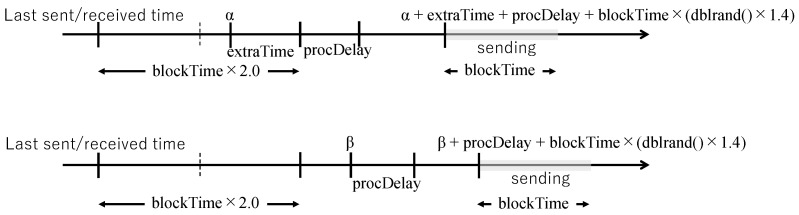
Packet transmission timing at sender and receiver nodes. Top: scheduling when the event occurs before blockTime×2.0 has elapsed since the last transmission/reception. Bottom: scheduling when the event occurs after blockTime×2.0 has elapsed.

Figure 9 shows packet transmission timing at sender and receiver nodes.

The transmission timing of coded packets (coded blocks) of data units in the Flood layer is explained. If the time α when a data unit is received from the application layer (for sender nodes) or when a coded block is received from the network layer (for receiver nodes) has not passed a certain time (blockTime×2.0) since the last transmission/reception time, transmission of the coded block set of the data unit is scheduled at the time obtained by adding the insufficient time extraTime and waiting time (procDelay+blockTime×(dblrand()×1.4)) to the reception time. The function dblrand() returns a uniformly distributed random double value in the range [0, 1). If the time β when a data unit or coded block is received has passed a certain time (blockTime×2.0) since the last transmission/reception time, transmission of the coded block set of the data unit is scheduled at the time obtained by adding the waiting time (procDelay+blockTime×(dblrand()×1.4)) to the reception time.

The processing delay is procDelay, and the transmission time of the coded block set of the data unit is blockTime. The transmission interval of the coded block set of the data unit is blockTime×(dblrand()×1.4+0.3).

## 8. Simulation Experiments Using OMNeT++ and Results

To evaluate the effectiveness of the proposed buffering and adaptive coding mechanisms, simulation experiments were conducted using OMNeT++ and the INET framework. The experiments compare flooding with fragmentation and reconstruction, flooding with RNC, and flooding with RNC incorporating the proposed mechanisms in VANET scenarios.

### 8.1. OMNeT++ Wireless Broadcast Network and Experimental Field

As wireless access technology, short-range communication in the 5.9 GHz band using Orthogonal Frequency Division Multiple Access (OFDM) methods, represented by IEEE 802.11p/WAVE and ETSI ITS-G5, is widely adopted, and vehicles distribute periodic status information (Cooperative Awareness Message: CAM, Basic Safety Message: BSM) to surrounding vehicles through one-hop broadcast. For applications requiring wide-area information dissemination, multi-hop information diffusion (flooding) based on retransmission control of broadcast packets is utilized. Meanwhile, in recent years, methods using cellular networks such as Cellular-V2X (C-V2X) and 5G NR-V2X have emerged, and further realization of high-reliability, low-latency communication is expected.

While IEEE 802.11p is mainstream in recent vehicle-mounted VANETs, this study uses IEEE 802.11b as the evaluation environment. This is because 802.11b is a widely adopted general-purpose wireless method and has high implementation ease and reproducibility in existing simulators and experimental equipment. Furthermore, in performance evaluation of broadcast communication and flooding methods, characteristics specific to ad hoc networks, such as contention, collision, and congestion, are the main factors rather than differences in bandwidth or modulation methods, so the validity for research purposes is not impaired by using 802.11b. Therefore, this study adopts IEEE 802.11b from the perspective of ensuring generality and comparability of evaluation.

For data transmission, IEEE 802.11b broadcast is used, assuming applications for information sharing between nodes in ad hoc networks. Regarding connectivity management between nodes in the wireless network, the carrier frequency is 2.412 GHz, the maximum output is 10 mW, the signal attenuation threshold is −100 dBm, and the path loss exponent is 3.5. Regarding IEEE 802.11b physical parameters, transmission delay is set to true, maximum allowable output is 10 mW, output is 10 mW, bit rate is 11 Mbps, and thermal noise is −100 dBm.

**Figure 10:**
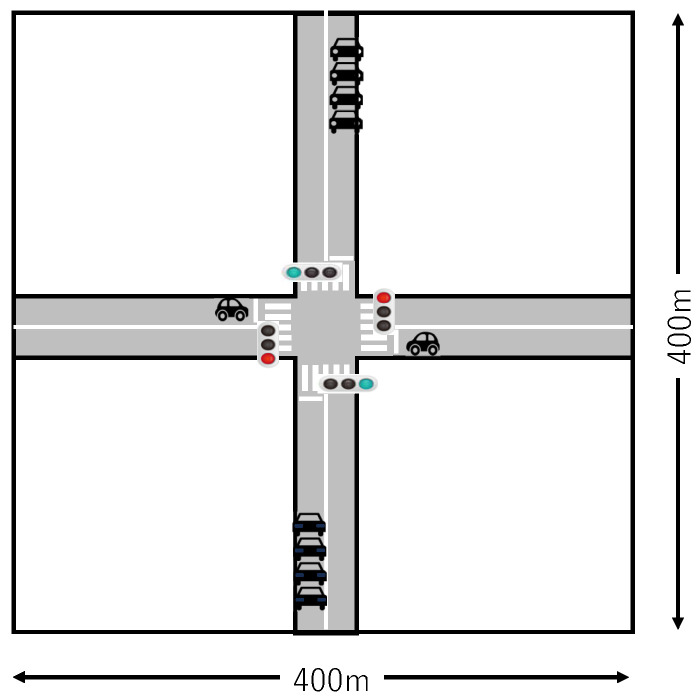
Vehicle-to-vehicle communication experimental field, including a single intersection.

Figure 10 shows a vehicle-to-vehicle communication experimental field, including a single intersection.

The field is a 400 m × 400 m plane (altitude 100 m). A 7 m wide road (two lanes on each side, 3.5 m width per lane) intersecting at the center of this field is assumed. Except for the roads, there are no structures that block or reflect radio waves. On the horizontal road, one vehicle on each side is placed 10 m behind the center of the intersection (at the stop line position), in a state stopped at a red light. On the vertical road, four vehicles each (at 10 m intervals) on the top and bottom are placed 90 m behind the center of the intersection, in a state traveling at 16.6 m/s ≈ 60 km/h (without acceleration) with a green light.

The simulation time is 15 s, corresponding to the period during which the four vehicles on top and bottom approach the intersection, pass each other, and pass through. In the 15-s simulation, the four vehicles on top, the four vehicles on bottom, and the two vehicles stopped at the intersection gradually approach, become most densely concentrated at the intersection, and then separate. The topology of vehicle-to-vehicle broadcast communication changes among the group of four vehicles on top (nodes 3, 4, 5, 6), the group of four vehicles on the bottom (nodes 7, 8, 9, 10), and the group of two vehicles stopped at the intersection (nodes 1, 2).

Table 2 and Table 3 summarize the simulation parameters used in our experiments, including common settings shared across all experiments and experiment-specific parameters.

Table 3 presents the parameters that vary across the three experiments, along with detailed explanations of specific parameter choices.

### 8.2. Experiments to Verify the Effect of Flooding with RNC

Flooding with fragmentation and reconstruction, and Flooding with RNC are simulated on OMNeT++. The following metrics are measured for each node: the number of data units with one or more blocks arrived at the early release timeout, the number of reconstructable/decodable data units, the number of forwarded data units, and the number of accepted data units at the receive timeout.

The number of hops (how many nodes the blocks of an arrived data unit passed through) is examined, and the maximum number of hops among the blocks of a data unit is defined as the hop count of that data unit. The number of data units with one or more blocks arrived at the early release timeout, and the number of reconstructable/decodable data units is measured separately for each hop count. Each experiment was conducted 10 times with independently varied random seeds (seeds 0–9), and all reported values represent the mean across these 10 independent runs. The relative ordering of performance between the compared approaches was consistent across all 10 runs without exception, confirming that the observed improvements are not artifacts of specific random conditions.

For both methods, the maximum number of buffers is 40, receive timeout recvTimeout = 2.0 s, maximum hop count is 5, number of blocks (generation size) numBlock=5, early release timeout relTimeout=0.3 s, average transmission interval of data units at the application layer is unitTime×1.7, unitTime=1.0 s, and average transmission interval of data unit block sets at the network layer is blockTime×1.7, blockTime=0.08 s. For Plain Flooding with fragmentation and reconstruction, the processing delay is procDelay=0.01 s. For flooding with RNC, the processing delay is procDelay=0.04 s.

The maximum number of buffers is set to 40 to ensure that all nodes can continuously transmit and receive without exceeding the maximum buffer count, i.e., to prevent the buffer discard policy from being applied.

Since RNC involves encoding and decoding processes, the processing delay procDelay for flooding with RNC is set larger, from 0.01 s to 0.04 s. The value procDelay=0.04 s is estimated as follows. The computational complexity of RNC encoding and decoding is approximately O(h·N) — roughly *h* times that of Plain Flooding (O(N)), where *h* is the generation size and *N* is the block data size — so a naive estimate gives 0.01×5=0.05 s. Accounting for CPU cache and parallel instruction effects that reduce the practical overhead below the theoretical *h*-fold increase, the effective multiplier is set to 4, yielding procDelay=0.01×4=0.04 s.

**Figure 11:**
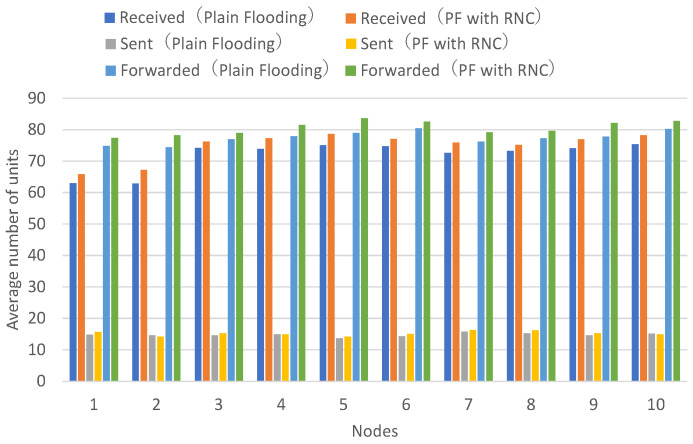
Comparison of Plain Flooding with Data Unit Fragmentation and Reconstruction (PF) and Plain Flooding with Randomized Network Coding (PF with RNC). Average number of received, sent, and forwarded data units for all nodes. Each value represents the mean across 10 independent runs.

**Figure 12:**
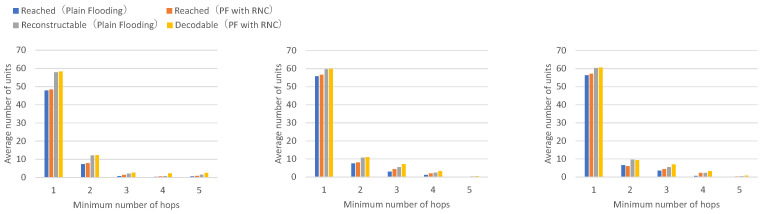
Per-hop results for vehicle groups comparing Plain Flooding (PF) and Plain Flooding with RNC (PF with RNC). Left: Intersection vehicles (Nodes 1, 2), Center: Top vehicles (Nodes 3, 4, 5, 6), Right: Bottom vehicles (Nodes 7, 8, 9, 10). Each value represents the mean across 10 independent runs.

Figure 11 presents the number of received units, transmitted units, and forwarded units for each node, showing that flooding with RNC achieves a higher number of received and forwarded units for approximately half of the nodes. Figure 12 presents the number of decodable (reconstructable) units per hop and arrived units per hop for each node, demonstrating that flooding with RNC achieves larger numbers of decodable (reconstructable) or arrived units at higher hop counts across all nodes.

Although flooding with RNC has a larger processing delay for encoding and decoding, resulting in smaller total numbers of received units and forwarded units, it can still be confirmed that the numbers of received units and forwarded units are improved for half of the nodes.

In flooding with RNC, when packet loss occurs on wireless paths to neighboring nodes, decoding becomes possible using coded packets obtained from other wireless paths. In other words, it can be confirmed that the arrival rate and acceptance rate of units that have passed through two or more hops are improved.

### 8.3. Experiments to Verify the Effect of Buffering in Flooding with RNC

For flooding with RNC, simulations are conducted on OMNeT++ comparing cases applying our proposed buffering versus other buffering methods. The following metrics are measured for each node: the number of data units with one or more blocks arrived at the early release timeout, the number of decodable data units, the number of forwarded data units, and the number of accepted data units at the receive timeout. The number of data units with one or more blocks arrived at the early release timeout, and the number of decodable data units is measured separately for each hop count.

The parameters are set as follows: maximum number of buffers is 25 or 40, receive timeout recvTimeout=2.0 s, maximum hop count is 5, number of blocks (generation size) numBlock=5, early release timeout relTimeout=0.3 s, average transmission interval of data units at the application layer is unitTime×1.7, unitTime=1.0 s, average transmission interval of data unit block sets at the network layer is blockTime×1.7, blockTime=0.08 s, and processing delay procDelay=0.04 s.

Regarding the FIFO discard policy, a comparison is made between when the maximum number of buffers (denoted as bcMax) is 40 and when it is 25. When the maximum number of buffers is 25, if all nodes are continuously sending and receiving, the maximum number of buffers will be exceeded, and the buffer discard policy will be applied.

**Figure 13:**
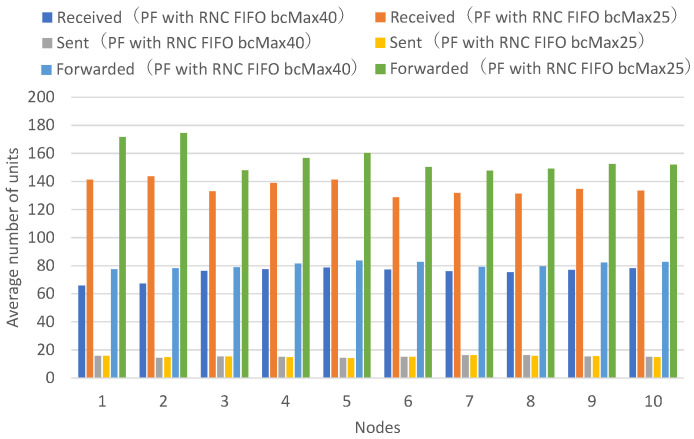
Comparison of a FIFO discard policy with a maximum of 40 buffers and a FIFO discard policy with a maximum of 25 buffers. Average number of received, sent, and forwarded data units for all nodes. Each value represents the mean across 10 independent runs.

**Figure 14:**
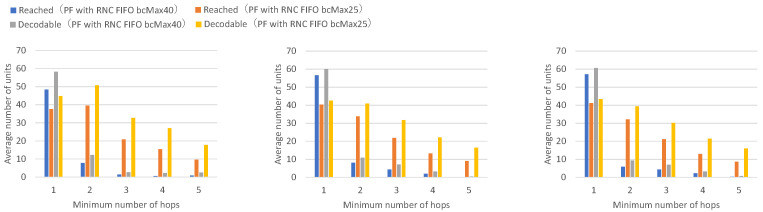
Per-hop results for vehicle groups comparing a FIFO discard policy with a maximum of 40 buffers and a FIFO discard policy with a maximum of 25 buffers. Left: Intersection vehicles (Nodes 1, 2), Center: Top vehicles (Nodes 3, 4, 5, 6), Right: Bottom vehicles (Nodes 7, 8, 9, 10). Each value represents the mean across 10 independent runs.

Figure 13 presents the numbers of received units, transmitted units, and forwarded units for each node, indicating that FIFO with a maximum of 25 buffers achieves higher numbers of received and forwarded units across all nodes. Figure 14 presents the numbers of decodable units per hop and arrived units per hop for each node, indicating higher numbers of decodable and arrived units across all nodes.

Among the forwarded unit counts for FIFO with a maximum of 25 buffers, some exceed the total number of transmitted units, indicating that the same data units are being forwarded multiple times. Specifically, the total number of data units transmitted across all nodes is 152.0. Even if all nodes could receive all units transmitted by every other node without loss, the maximum receivable units per node would be at most 136.8 on average (152.0 minus own transmission). The forwarded unit counts for FIFO with a maximum of 25 buffers exceed this theoretical maximum for all nodes, by 8.2% to 27.0%, confirming that duplicate forwarding is occurring. Furthermore, the ratio of forwarded to sent units ranges from 9.1 to 11.9 (mean: 10.3) for FIFO with a maximum of 25 buffers, compared to 4.9 to 5.9 (mean: 5.3) for FIFO with a maximum of 40 buffers.

With FIFO with a maximum of 40 buffers, the maximum buffer count is not exceeded even when all nodes continuously transmit and receive, allowing individual buffers to be retained until the receive timeout expires without premature discard. With FIFO with a maximum of 25 buffers, when all nodes continuously transmit and receive, the maximum buffer count is exceeded, so buffers created earlier are successively discarded by buffers for newly arriving units, and buffers are not maintained until the receive timeout expires. This prevents sufficient coded blocks from accumulating in individual buffers for successful decoding.

With FIFO with a maximum of 25 buffers, buffers are discarded in a short time, causing the same unit to be repeatedly received and forwarded, and the number of received units and forwarded units increases by the amount of duplicate units. It can be confirmed that the FIFO discard policy wastes network bandwidth through repeated reception and forwarding of the same unit when the number of nodes is large or when the unit transmission rate is high.

Next, a comparison is made between the FIFO discard policy when the maximum number of buffers is 40, and the LIFO discard policy when the maximum number of buffers is 25.

**Figure 15:**
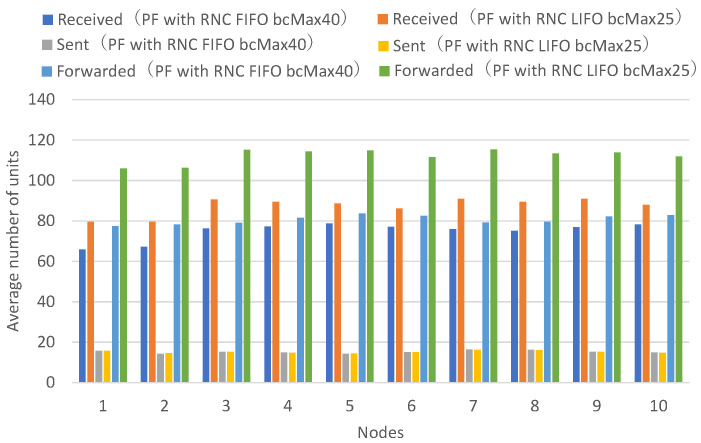
Comparison of a FIFO discard policy with a maximum of 40 buffers and a LIFO discard policy with a maximum of 25 buffers. Average number of received, sent, and forwarded data units for all nodes. Each value represents the mean across 10 independent runs.

**Figure 16:**
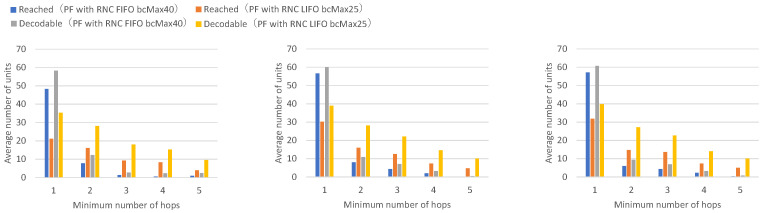
Per-hop results for vehicle groups comparing a FIFO discard policy with a maximum of 40 buffers and a LIFO discard policy with a maximum of 25 buffers. Left: Intersection vehicles (Nodes 1, 2), Center: Top vehicles (Nodes 3, 4, 5, 6), Right: Bottom vehicles (Nodes 7, 8, 9, 10). Each value represents the mean across 10 independent runs.

Figure 15 presents the numbers of received units, transmitted units, and forwarded units for each node, indicating that LIFO with a maximum of 25 buffers achieves higher numbers of received and forwarded units across all nodes. Figure 16 presents the numbers of decodable units per hop and arrived units per hop for each node, indicating higher numbers of decodable and arrived units for most nodes.

With FIFO with a maximum of 40 buffers, individual buffers are retained until the receive timeout expires. With LIFO, with a maximum buffer count of 25, except for the most recently created buffer, other buffers are retained until the receive timeout expires. Buffers for newly arriving units are not created until existing buffers are discarded after the receive timeout expires.

With LIFO, with a maximum of 40 buffers, newly arriving units are not accepted unless there is sufficient capacity in the number of existing buffers; thus, when many units arrive from neighboring nodes, the forwarding rate is suppressed below a certain level, and the numbers of received units and forwarded units increase by the amount that does not congest the network bandwidth. It can be confirmed that the LIFO discard policy, in addition to preventing repeated reception and forwarding of the same unit, reduces the load on network bandwidth in downstream unit forwarding, excluding the effects of unit transmission and forwarding rates from neighboring nodes.

Next, a comparison is made between the LIFO discard policy when the maximum number of buffers is 25, and the LIFO+LRU discard policy when the maximum number of buffers is 25.

**Figure 17:**
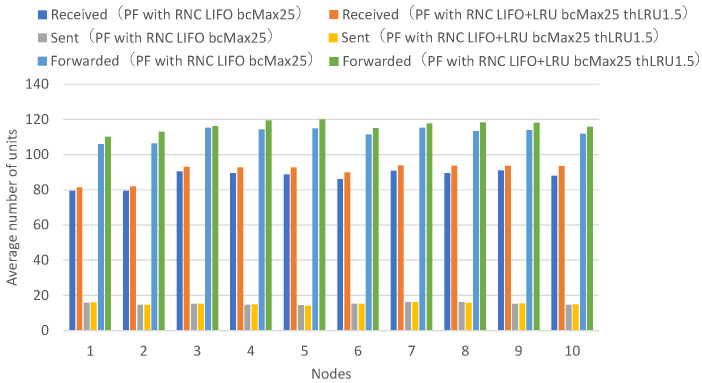
Comparison of a LIFO discard policy with a maximum of 25 buffers and a LIFO+LRU discard policy with a maximum of 25 buffers. Average number of received, sent, and forwarded data units for all nodes. Each value represents the mean across 10 independent runs.

**Figure 18:**
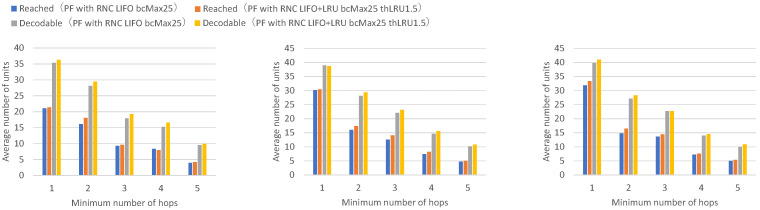
Per-hop results for vehicle groups comparing a LIFO discard policy with a maximum of 25 buffers and a LIFO+LRU discard policy with a maximum of 25 buffers. Left: Intersection vehicles (Nodes 1, 2), Center: Top vehicles (Nodes 3, 4, 5, 6), Right: Bottom vehicles (Nodes 7, 8, 9, 10). Each value represents the mean across 10 independent runs.

Figure 17 presents the numbers of received units, transmitted units, and forwarded units for each node, indicating that LIFO+LRU with a maximum of 25 buffers achieves higher numbers of received and forwarded units for most nodes. Figure 18 presents the numbers of decodable units per hop and arrived units per hop for each node, indicating higher numbers of decodable and arrived units for most nodes.

With LIFO and a maximum buffer count of 25, some existing buffers containing incomplete units may have a low probability of receiving additional blocks. Discarding such buffers creates space to accept new units. With LIFO+LRU with a maximum buffer count of 25, among buffers with incomplete units where the elapsed time since the last block arrival exceeds a threshold (thLRU=1.5 s), the one with the longest elapsed time is discarded.

With LIFO+LRU with a maximum buffer count of 25, buffers with a low probability of future block arrivals are discarded, and the numbers of received units and forwarded units increase by the number of accepted new units. It can be confirmed that the LIFO+LRU discard policy, in addition to the effects of the LIFO discard policy, improves the ability to accept new units, i.e., improves the arrival rate and acceptance rate of units.

### 8.4. Experiments to Verify the Effect of Adaptive Coding in Flooding with RNC

For flooding with RNC (LIFO+LRU discard policy), simulations are conducted on OMNeT++ comparing cases with and without applying our proposed adaptive coding. The following metrics are measured for each node: the number of data units with one or more blocks arrived at the early release timeout, the number of decodable data units, the number of forwarded data units, and the number of accepted data units at the receive timeout. The number of data units with one or more blocks arrived at the early release timeout, and the number of decodable data units are measured separately for each hop count.

The parameters are set as follows: maximum number of buffers is 25, receive timeout recvTimeout=2.0 s, maximum hop count is 5, number of blocks (generation size) numBlock=5, early release timeout relTimeout=0.3 s, average transmission interval of data units at the application layer is unitTime×1.7, unitTime=1.0 s, average transmission interval of data unit block sets at the network layer is blockTime×1.7, blockTime=0.16 s, processing delay procDelay=0.04 s, and time threshold for applying LRU thLRU=1.8 s. When applying our adaptive coding, lB=5, uB=8,10,12, and dTh=1.0.

In adaptive coding, since the number of coded packets is varied in the range lB to uB, the transmission time of data unit block sets blockTime is set larger, from 0.08 s to 0.16 s. To avoid giving an advantage to the case with adaptive coding applied, blockTime=0.16 s is similarly set for the case without adaptive coding applied.

When the number of blocks of data units arriving at a node continues to be insufficient, i.e., when the average duplication rate is below 1.0, it is considered appropriate to increase the number of encoded packets, and the duplication rate threshold dTh=1.0 is set.

For the LIFO+LRU discard policy when the maximum number of buffers is 25, a comparison is made between adaptive coding that varies the number of encoded packets between 5 and 8 (denoted as uB) and adaptive coding that varies the number of encoded packets between 5 and 10.

**Figure 19:**
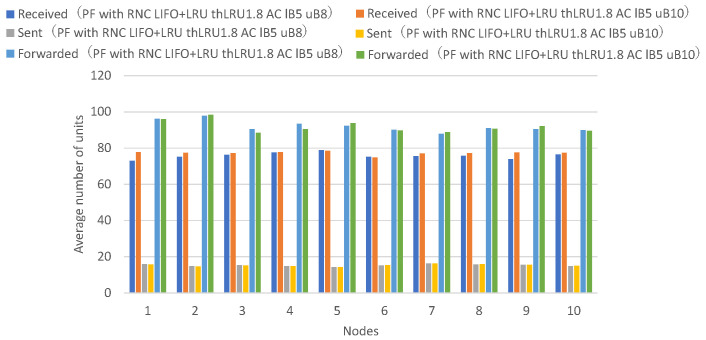
Comparison of adaptive coding with a minimum of 5 and maximum of 8 coded packets and adaptive coding with a minimum of 5 and maximum of 10 coded packets. Average number of received, sent, and forwarded data units for all nodes. Each value represents the mean across 10 independent runs.

**Figure 20:**
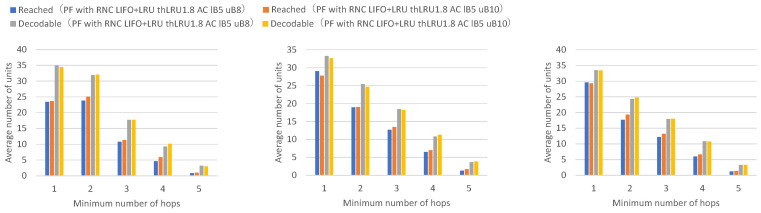
Per-hop results for vehicle groups comparing adaptive coding with a minimum of 5 and maximum of 8 coded packets and adaptive coding with a minimum of 5 and maximum of 10 coded packets. Left: Intersection vehicles (Nodes 1, 2), Center: Top vehicles (Nodes 3, 4, 5, 6), Right: Bottom vehicles (Nodes 7, 8, 9, 10). Each value represents the mean across 10 independent runs.

Figure 19 presents the numbers of received units, transmitted units, and forwarded units for each node, indicating that adaptive coding with a lower bound of 5 and an upper bound of 10 on the number of coded packets achieves higher numbers of received and forwarded units for many nodes. Figure 20 presents the numbers of decodable units per hop and arrived units per hop for each node, indicating higher numbers of decodable and arrived units for many nodes.

Adaptive coding with an upper bound of 10 for the number of coded packets shows higher numbers of units arrived at 2 or more hops and decodable units by the amount of additional forwarded coded packets compared to adaptive coding with an upper bound of 8.

Next, a comparison is made between adaptive coding that varies the number of encoded packets between 5 and 10 and adaptive coding that varies the number of encoded packets between 5 and 12.

**Figure 21:**
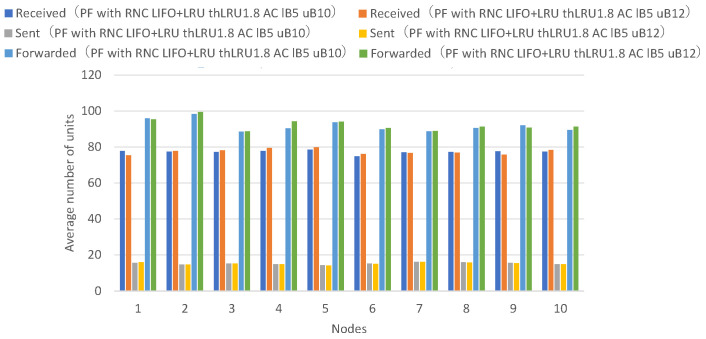
Comparison of adaptive coding with a minimum of 5 and maximum of 10 coded packets and adaptive coding with a minimum of 5 and maximum of 12 coded packets. Average number of received, sent, and forwarded data units for all nodes. Each value represents the mean across 10 independent runs.

**Figure 22:**
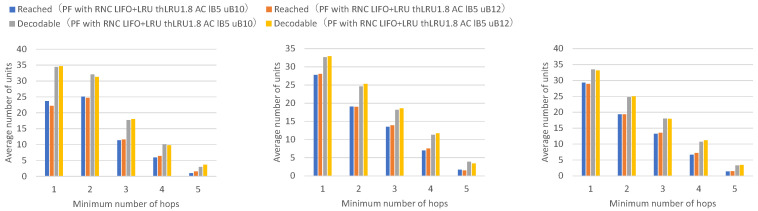
Per-hop results for vehicle groups comparing adaptive coding with a minimum of 5 and maximum of 10 coded packets and adaptive coding with a minimum of 5 and maximum of 12 coded packets. Left: Intersection vehicles (Nodes 1, 2), Center: Top vehicles (Nodes 3, 4, 5, 6), Right: Bottom vehicles (Nodes 7, 8, 9, 10). Each value represents the mean across 10 independent runs.

Figure 21 presents the numbers of received units, transmitted units, and forwarded units for each node, indicating that adaptive coding with a lower bound of 5 and an upper bound of 12 on the number of coded packets achieves higher numbers of received units for many nodes, while only a few nodes exhibit higher numbers of forwarded units. Figure 22 presents the numbers of decodable units per hop and arrived units per hop for each node, indicating that many nodes achieve higher numbers of decodable and arrived units at higher hop counts.

Adaptive coding with an upper bound of 12 for the number of coded packets shows higher numbers of units arrived at 2 or more hops and decodable units by the amount of additional forwarded coded packets compared to adaptive coding with an upper bound of 10. However, the amount of increase is smaller compared to the comparison between upper bound 8 and upper bound 10, indicating diminishing marginal improvements.

By increasing the upper bound of the number of coded packets, the number of units arrived at 2 or more hops, and the number of decodable units increases further. It can be confirmed that the upper bound that can be set has a limit, as the number of coded packets that can be forwarded at once in the network depends on the average transmission interval of unit block sets, which constrains achievable redundancy levels.

Next, a comparison is made between no adaptive coding and adaptive coding with varying the number of coded packets in the range of 5 to 12 for a LIFO+LRU discard policy when the maximum number of buffers is 25.

**Figure 23:**
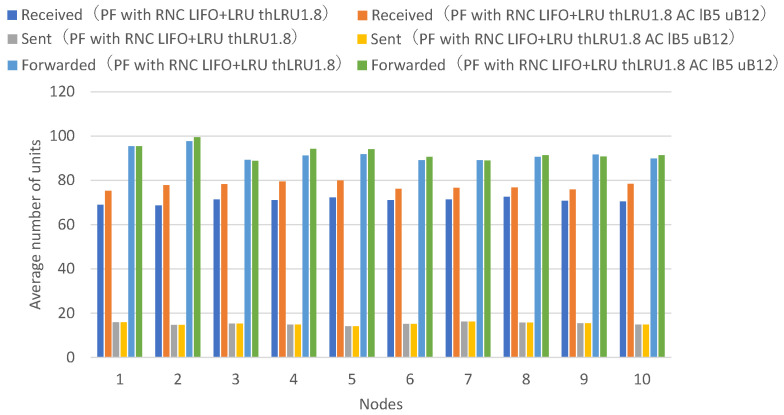
Comparison of Flooding with RNC without adaptive coding and adaptive coding with a lower limit of 5 and an upper limit of 12 coded packets. Average number of received, sent, and forwarded data units for all nodes. Each value represents the mean across 10 independent runs.

**Figure 24:**
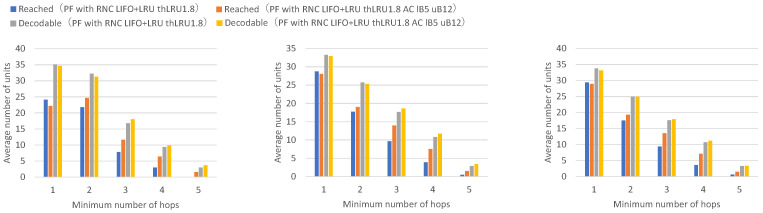
Per-hop results for vehicle groups comparing Flooding with RNC without adaptive coding and adaptive coding with a lower limit of 5 and an upper limit of 12 coded packets. Left: Intersection vehicles (Nodes 1, 2), Center: Top vehicles (Nodes 3, 4, 5, 6), Right: Bottom vehicles (Nodes 7, 8, 9, 10). Each value represents the mean across 10 independent runs.

Figure 23 presents the numbers of received units, transmitted units, and forwarded units for each node, indicating that adaptive coding with a lower bound of 5 and an upper bound of 12 on the number of coded packets achieves higher numbers of received units across all nodes. Figure 24 presents the numbers of decodable units per hop and arrived units per hop for each node, indicating that most nodes achieve higher numbers of decodable and arrived units.

In our adaptive coding, by making the number of coded packets larger than the generation size, redundant coded packets are created, and even if packet loss occurs on a wireless path to a certain node, the probability increases that coded blocks can be obtained up to the generation size from the same wireless path, thereby improving decoding success rates. This increases resilience against packet loss in mobile ad hoc networks.

Although there is a limit to the upper bound of the number of coded packets, depending on the average transmission interval of unit block sets, it was confirmed that the number of decodable units and arrived units at 2 or more hops increases, and as a result, the number of received units increases, particularly in multi-hop scenarios experiencing severe packet loss.

These experimental results demonstrate that the proposed LIFO+LRU buffering policy prevents premature buffer discard and the proposed adaptive coding mechanism increases transmission redundancy when needed, thereby improving the arrival rate and acceptance rate of data units in flooding with randomized network coding under the broadcast storm problem.

## 9. Conclusions

This paper addressed the broadcast storm problem in flooding via wireless broadcast for many-to-many communication in wireless ad hoc networks. We proposed a novel buffering mechanism and adaptive coding strategies to improve data unit reception rates in flooding with randomized network coding (RNC), thereby mitigating the impact of packet loss caused by broadcast storms.

To validate the effectiveness of our proposals, we implemented flooding protocols using the discrete event simulator OMNeT++ 6.2.0 and the INET framework 4.5.4, which are suitable for integrated experiments involving networks, applications, and the surrounding physical systems. We conducted simulation experiments assuming Vehicular Ad Hoc Networks (VANETs) with LinearMobility patterns and IEEE 802.11b wireless specifications. Three protocols were implemented at the network layer by modifying the Flood layer components (Flood.h, Flood.cc, Flood.ned) and network layer components (Chunk, Tag): (1) Plain Flooding with data unit fragmentation and reconstruction, (2) Plain Flooding with RNC, and (3) Plain Flooding with RNC incorporating our proposed buffering and adaptive coding mechanisms, all including processing delays and collision avoidance.

Regarding buffering mechanisms used during decoding and forwarding processes, we proposed a buffer discard policy combining Last-In-First-Out (LIFO) and Least Recently Used (LRU) strategies for managing coded blocks associated with data units received from multiple neighboring nodes. The proposed discard policy prioritizes discarding incomplete data unit buffers where the elapsed time since the last coded block arrival exceeds a threshold and represents the longest idle period when multiple buffers exist, and the buffer limit is reached. If no such buffer exists, the most recently created buffer is discarded following the LIFO principle.

Experimental results demonstrated that the FIFO discard policy causes premature buffer discard when receiving coded blocks associated with numerous different data units from multiple neighboring nodes. Since individual buffers are not retained for extended periods, the probability of receiving all coded blocks of a data unit decreases, resulting in repeated reception of the same data unit. The LIFO discard policy retains individual buffers until the receive timeout expires, regardless of data unit transmission patterns from neighboring nodes, increasing the number of accepted data units and preventing redundant reception through timeout adjustment. Furthermore, combining LIFO and LRU policies by discarding stale buffers unlikely to receive additional coded blocks further increases the number of accepted data units, as confirmed experimentally.

Regarding adaptive coding, we proposed dynamically adjusting the number of coded packets generated during coding and forwarding processes after decoding, without requiring inter-node coordination, based on the data unit reception status at each node. The proposed adaptive coding maintains a fixed generation size while calculating the duplication rate of data units arriving at the node and increasing or decreasing the number of coded packets depending on whether this rate exceeds a threshold. The duplication rate is defined as the average number of times complete data units are received in duplicate and is updated when buffers exceeding the discard time are removed.

In wireless ad hoc networks, where the number of neighboring nodes and link states change frequently, approaches that modify the generation size require node coordination and become complex. Our adaptive coding allows each node to independently vary the number of coded packets based on the duplication rate without coordination, adjusting only through wireless broadcast. Experimental results confirmed that when complete data units are difficult to receive at a node, adaptive coding enhances RNC effectiveness and increases the number of accepted data units. While increasing the number of coded packets enhances RNC effects, it consumes additional network bandwidth. Experiments confirmed that increasing the maximum number of coded packets, dependent on the transmission time of the coded block set, progressively increases the number of accepted data units.

Our contributions include novel protocol implementations for OMNeT++ at the network layer and validated strategies demonstrating the effectiveness of LIFO and LIFO+LRU buffering policies and adaptive coding for improving RNC-based flooding in mobile wireless ad hoc networks through VANET simulation experiments. The proposed mechanisms require no inter-node coordination, making them practical for dynamic mobile ad hoc network environments where topology changes frequently.

Future work includes evaluating the proposed mechanisms under various network sizes and densities, mobility patterns, and traffic conditions, as well as investigating their applicability to other wireless ad hoc network scenarios beyond VANETs.

## Figures and Tables

**Table 1:** Comparison with related work.

Work	Communication Model	Feedback Required	Adaptation Method	Environment	Implementation
Matsuda et al. [4]	Many-to-many broadcast	No	Fixed parameters	Dense ad hoc	Simulation
Vu et al. [5]	Combined unicast/ broadcast	Unicast ACK	Link loss estimation	MANET	Simulation
Yoshida et al. [6]	Many-to-many broadcast	No	Node mobility (local)	VANET	Simulation
Zhang et al. [7]	Many-to-one	No	Fixed batch	V2V theoretical	Analysis and Simulation
Tetrys [8]	Point-to-point	Unicast ACK	Sliding window	Streaming	Experimental
Zhang et al. [9]	Point-to-point	Unicast ACK	Reinforcement learning	Maritime IoT	Simulation
**Our work**	**Many-to-many broadcast**	**No**	**Duplication rate (local)**	**VANET flooding**	**OMNeT++ simulation**

**Table 2:** Simulation parameters (common settings).

Category	Parameter	Value
*Simulation Environment*		
	Simulator/Framework	OMNeT++ 6.2.0/INET 4.5.4
	Total simulation time	15 s
	Warm-up period	2 s (excluded from analysis)
	Measurement period	13 s (seconds 2–15)
	Number of runs	10 (per configuration)
	Random seeds	0–9 (systematically varied)
	Reported values	Mean across 10 runs
*Experimental Field*		
	Field size	400 m × 400 m
	Road configuration	7 m wide, intersection
	Number of vehicles	10 (2 stopped, 8 moving)
	Initial distance	90 m from intersection center
	Vehicle spacing	10 m
	Velocity	16.6 m/s (60 km/h)
*Physical Layer*		
	Standard	IEEE 802.11b
	Carrier frequency	2.412 GHz
	Bit rate	11 Mbps
	Tx power	10 mW
	Receiver sensitivity	−100 dBm
	Path loss exponent	3.5
	Communication range	∼100 m
	Thermal noise	−100 dBm
*Common Protocol Parameters*		
	Maximum hop count	5
	Generation size (*h*)	5 blocks
	Symbol length	8 bits (GF($2^{8}$))
	Early release timeout	0.3 s
	Receive timeout	2.0 s
	unitTime	1.0 s

**Table 3:** Simulation parameters (experiment-specific settings).

Parameter	Exp. 1 (Section 8.2) Effect of RNC	Exp. 2 (Section 8.3) Effect of Buffering	Exp. 3 (Section 8.4) Effect of Adaptive Coding
procDelay (non-RNC)	0.01 s	—	—
procDelay (RNC)	0.04 s	0.04 s	0.04 s
blockTime	0.08 s ^*a*^	0.08 s ^*a*^	0.16 s ^*b*^
bcMax	40 ^*c*^	25 or 40	25
Discard policy	— ^*c*^	FIFO/LIFO/LIFO+LRU	LIFO+LRU
thLRU	—	1.5 s ^*d*^	1.8 s ^*d*^
Coded packets	Fixed: 5	Fixed: 5	lB=5, uB∈{8,10,12}
dTh	—	—	1.0

^*a*^ blockTime is set to 0.08 s in Experiments 1 and 2 because the number of coded packets are fixed at 5 (equal to the generation size *h*) for all compared approaches in these experiments. ^*b*^ blockTime is set to 0.16 s (doubled) in Experiment 3 to accommodate the variable number of coded packets in adaptive coding (up to uB=12). To ensure a fair comparison, the same blockTime value is applied to the case without adaptive coding in this experiment. ^*c*^ In Experiment 1, bcMax is set to 40 to ensure that all nodes can continuously transmit and receive without exceeding the maximum buffer count, i.e., to prevent the buffer discard policy from being triggered during the experiment. ^*d*^ thLRU is used only when the LIFO+LRU discard policy is applied. The value is determined based on the maximum propagation delay across 5 hops: blockTime×2+(maxHop−1)×(blockTime×2+procDelay), rounded up to the nearest 0.1 s, subject to the constraint thLRU<recvTimeout=2.0 s. For Exp. 2 (blockTime=0.08 s): 0.08×2+4×(0.08×2+0.04)=0.96 s ≈1.0 s. A margin of 0.5 s is added to account for variability in coded block arrival timing, giving thLRU=1.0+0.5=1.5 s. For Exp. 3 (blockTime=0.16 s): 0.16×2+4×(0.16×2+0.04)=1.76 s ≈1.8 s. No margin is added, as adding 0.5 s would exceed recvTimeout=2.0 s, giving thLRU=1.8 s.

## Data Availability

The original contributions presented in this study are included in the article. Further inquiries can be directed to the corresponding author.
